# A novel peptide therapeutic targeting PD1 immune checkpoint with equipotent antagonism of both ligands and a potential for better management of immune-related adverse events

**DOI:** 10.1186/2051-1426-1-S1-O24

**Published:** 2013-11-07

**Authors:** Pottayil Sasikumar, Rajeev Shrimali, Sreenivas Adurthi, Raghuveer Ramachandra, Leena Satyam, Amit Dhudashiya, Dodheri Samiulla, K B Sunilkumar, Murali Ramachandra

**Affiliations:** 1 Aurigene Discovery Technologies, Bangalore, India

## 

Recent advances in achieving highly durable clinical responses via inhibition of immune checkpoint proteins including CTLA-4 and PD1 have revolutionized the outlook for cancer therapy. However, along with impressive clinical activity (response rate of ~25% with either anti-CTLA-4 or anti-PD1 as single agent, but > 50% with a combination), severe immune-related adverse events (irAEs) due to the breaking of immune self- tolerance (25-30% with anti-CTLA-4 and up to 15-17% with anti-PD1) are becoming increasingly evident. Sustained target inhibition as a result of a long half-life (>15-20 days) and >70% target occupancy for months are likely contributing to severe irAEs observed in the clinic with antibodies targeting immune checkpoint proteins. Our efforts are therefore focused on developing immune checkpoint blockers with potent anti-tumor activity but with a shorter pharmacokinetic profile as a strategy to better manage severe irAEs. Flexibility in adjusting the drug exposure because of a shorter t½ could also be advantageous for use in combination with other checkpoint modulators or anti-cancer agents.

Peptide antagonist AUR-012, constructed with elements from human PD-1, displayed equipotent antagonism towards PD-L1 and PD-L2 with potent activity in rescue of lymphocyte proliferation and effector functions. Rescue of proliferation of immune cells analyzed upon stimulation with anti-CD3/anti-CD-28 indicated a complete rescue of CD4+ and CD8+ T cells. Interestingly, the proliferation of CD4+, Foxp3+ T cells was completely abolished with AUR-012 treatment indicating a complete suppression of regulatory T cells. Sustained activation of circulatory immune cells and their ability to secrete IFN-γ up to 72 h indicate that pharmacodynamic effects persist even after the clearance of AUR-012 in animal models, thus supporting a dosing interval of up to 3 days. In models of melanoma, breast, kidney and colon cancers, AUR-012 showed efficacy in inhibition of both primary tumor growth and metastasis. Additionally, anti-tumor activity of AUR-012 in a pre-established CT26 model correlated well with pharmacodynamic effects as indicated by intratumoral recruitment of CD4+ and CD8+ T cells, and a reduction in PD1+ T cells (both CD4+ & CD8+) in tumor and blood. In 14-day repeated dose toxicity studies, AUR -012 was well tolerated at 100x of the efficacious doses.

These findings demonstrating equipotent antagonism of both PD-L1 and PD-L2 signaling and the observed correlation between anti-tumor activities with the modulation of specific T-cell populations support further development of AUR-012 in the clinic.

